# Microencapsulation protects the biological activity of sea buckthorn seed oil

**DOI:** 10.3389/fnut.2022.1043879

**Published:** 2023-01-11

**Authors:** Huirong Zhang, Guanjie Song, Wenrui Ma, Miaomiao Guo, Xiao Ling, Dan Yu, Weiqiang Zhou, Li Li

**Affiliations:** ^1^College of Chemistry and Materials Engineering, Beijing Technology and Business University, Beijing, China; ^2^Beijing Key Laboratory of Plants Resource Research and Development, Beijing, China; ^3^Beijing Lan Divine Technology Co., Ltd., Beijing, China; ^4^Nutrition and Health Research Institute, COFCO Corporation, Beijing, China

**Keywords:** *Hippophae rhamnoides* seed oil, spray-drying method, GC-MS, oxidation stability, antioxidant activity, microcapsule

## Abstract

**Introduction:**

Sea buckthorn (*Hippophae rhamnoides*) seed oil is rich in unsaturated fatty acids, and is thus susceptible to oxidation and rancidity. Microencapsulation technology allows the effective protection of active substances, thereby prolonging the deterioration time and shelf life.

**Methods:**

In this study, *H. rhamnoides* microcapsules were prepared using a spray-drying method, and the microencapsulation parameters were optimized. The morphological characteristics, structural parameters, and stability of the microcapsules were determined using scanning electron microscopy, Fourier transform infrared spectroscopy, thermogravimetric analysis, differential scanning calorimetry, and oil oxidation stability testing.

**Results:**

Based on encapsulation efficiency (EE, %) and the particle size (D50) of the microcapsules, the optimal preparation conditions were characterized as a wall material consisting of soy protein isolate and soybean polysaccharide (2:3), a wall concentration of 15%, a core-to-wall ratio of 1:3, and an inlet temperature of 160°C. Under these optimal conditions, the encapsulation efficiency was 95.30 ± 2.67%, with a yield of 57.03 ± 3.71% and a particle size of 7.96 ± 1.04 μm.

**Discussion:**

Furthermore, the effectiveness of microencapsulation in protecting the biological activity of *H. rhamnoides* seed oil was confirmed by an antioxidation test. Thus, the results of this study showcase the successful microencapsulation of *H. rhamnoides* seed oil, thereby significantly improving its stability.

## 1. Introduction

Sea buckthorn (*Hippophae rhamnoides* L.) is a species of small shrub, from the genus Hippophae of the family Hojicaceae (1, 2). They are native to the mountainous areas of Asia and northwestern Europe (3). China has the richest germplasm resources for *H. rhamnoides* plants, with north and southwest China having the highest density of *H. rhamnoides* worldwide (4, 5).

The seeds of the *H. rhamnoides* fruit are inedible and account for 23% (w/w) of the weight, with an oil content of 10.0–16.3% (6). But seed oil is rich in unsaturated fatty acid (UFAs, 71.2–76.0%). Mainly includes oleic acid (OA), linoleic acid (LA) and linolenic acid (ALA). OA, a monounsaturated fatty acid, can reduce the risk of cardiovascular disease by lowering serum triglyceride (TG) levels (7, 8). The polyunsaturated fatty acids LA and ALA are essential fatty acids. LA has the ability to lower serum cholesterol levels and inhibit arterial thrombosis, which is good for the prevention of cardiovascular and cerebrovascular diseases (9). ALA plays an important role in the development of brain and vision in infants and children, and in the maintenance of normal blood lipid levels in adults (10, 11). The content and composition of OA, LA, and ALA are important indicators for evaluating the nutritional value of edible oils. The ratio of OA: LA: ALA in *H. rhamnoides* seed oil is about 1:4:2 (12). In addition to unsaturated fatty acids, *H. rhamnoides* seed oil contains various other bioactive components, such as carotenoids, vitamins, polyphenols, and flavonoids, that show antioxidant, antibacterial, antiviral, and immunity-boosting properties (13–15). This makes *H. rhamnoides* seed oil a highly promising functional edible oil.

However, because of the high content of UFAs, *H. rhamnoides* seed oil is susceptible to oxidation and souring when exposed to air, consequently having a short shelf life (16, 17). Oxidation damages the organoleptic taste, reduces the beneficial efficacy, and generates peroxides and free radicals that can cause cell damage and disease (18, 19). Therefore, the protection of *H. rhamnoides* seed oil from oxidation is paramount for its application.

Microencapsulation technology is an effective way to protect UFAs from oxidation by using polymer materials to form a protective shell. The shell encapsulates the oil particles, forming a barrier against air, thus reducing the contact between the oil and oxygen, thereby preventing oil deterioration (20, 21). Spray drying is a widely applicable method for the preparation of microcapsules because of its high production efficiency, good encapsulation effect, uniform size of the resulting microcapsules, and simple operation (22, 23). During spray drying, the conditions for emulsion preparation and spray drying directly affect the quality of the microcapsules.

Therefore, the main aim of this study was to investigate the effect of microencapsulation in protecting the biological activity of *H. rhamnoides* seed oil. The preparation parameters were optimized to obtain *H. rhamnoides* seed oil microcapsules *via* spray drying. The encapsulation efficiency (EE, %), particle size (D50), structural morphology, fatty acid composition, oxidative stability and antioxidant activity of the microcapsules were further investigated. Under the optimal process conditions, microencapsulation could effectively improve the oxidative stability and maintain the high biological activity of the oil. Microencapsulated *H. rhamnoides* seed oil with a long shelf life may have a wide application potential in medicine, food, cosmetics, and many other fields.

## 2. Experimental

### 2.1. Materials

*Hippophae rhamnoides* seed oil was obtained from Jiangxi Natural Fragrance Co., Ltd. BR grade whey protein (WPI), chitosan (CS), maltodextrin (MD), soy protein isolate (SPI), soy polysaccharide (SPS), and gum arabic (GA) were purchased from Shanghai Yuanye Biotechnology Co., Ltd. Sodium hydroxide, 2,2-diphenyl-1-picrylhydrazyl (DPPH), 2,2-azinobis (3-ethylbenzo-thiazoline-6-sulphonic acid) (ABTS), anhydrous ethanol, potassium persulfate (K_2_S_2_O_8_), n-hexane, and methanol were purchased from Shanghai Aladdin Biochemical Technology Co., Ltd. Boron trifluoride-methanol solution (14%) was purchased from Shanghai Macklin Biochemical Co., Ltd.

### 2.2. Preparation of microcapsules

The emulsions were prepared according to the wall material listed in Table 1, i.e., wall material A was added to 60.0 g of water and dissolved under heating at 60°C to obtain solution A; wall material B was dissolved in 28 g of water to obtain solution B. Solution A was homogenized at 10,000 rpm using an immersion disperser (KINEMATICA POLYTRON^®^ PT 2500E). Thereafter, *H. rhamnoides* seed oil (3.0 g) was slowly added to solution A and homogenized for 5 min, and solution B was then introduced and further homogenized for 5 min to obtain the emulsion. The spray dryer (Laiheng Science and Technology Co., Ltd L-217) was set at a peristaltic pump rate of 60% to spray dry the emulsion. The resulting *H. rhamnoides* seed oil microcapsule powder was collected from the separator.

**TABLE 1:** Selection of wall material combinations and proportions.

No.	Wall material and proportion (A:B)	Ratio (A:B)
1	WPI:CS	1:2
2	WPI:MD	1:2
3	SPI:SPS	1:2
4	SPI:GA	1:2
5	SPI:MD	1:2

The optimal process for the preparation of microcapsules was determined by comparing the effect of wall ratio (material A to B = 1:3, 1:2, 2:3, and 2:1), concentration of wall material (9.0, 12.0, 15.0, and 18.0%), core-to-wall ratio (1:5, 1:4, 1:3, and 2:5), and spray drying inlet temperature (140, 160, and 180°C) on the microcapsules.

### 2.3. Determination of encapsulation efficiency and yields

**FIGURE 1:**
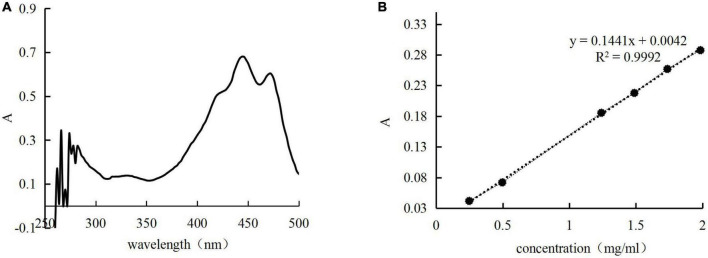
Full wavelength scan **(A)** and standard curve **(B)** for n-hexane solution of *Hippophae rhamnoides* seed oil.

A standard curve was created at the maximum absorption wavelength (444 nm) to calculate the concentration of the *H. rhamnoides* seed oil (Figure 1).

Two identical amounts of microcapsules were dissolved in hexane, one was shaken, and the other was shaken and sonicated for 1 h to obtain supernatant solutions. The absorbance of the supernatant solutions was measured separately to calculate the surface oil (SO) and total oil (TO) contents. Following this, the EE was calculated using the formula:u


$$\text{E}\,E=\left(1-\frac{\text{S}\,O}{\text{T}\,O}\right)\times 100\%$$

Microcapsules in the separator were collected, and the microcapsule yield was calculated using the following equation:


$$\text{Y}=\frac{\text{M}}{\text{m}}\times 100\%$$

where *M* is the mass of the microcapsules and *m* is the total mass of the seed oil and wall material.

### 2.4. Microcapsules particle size measurement

The microcapsules were re-dissolved in water to obtain a homogeneous emulsion, and the D50 of the emulsion was determined using a Malvern Mastersizer 3000 laser diffraction particle size analyzer. D50 was recorded as the particle size at 50% of the cumulative size in the distribution curve.

### 2.5. Fourier transform infrared spectroscopy (FT-IR)

The samples for FT-IR were prepared by the compression method. Sample aliquots (2.0 mg of *H. rhamnoides* seed oil, SPS, SPI, and microcapsules) were mixed with 198.0 mg of potassium bromide (KBr) in a mortar, ground well, and then pressed into clear flakes for testing by FT-IR (PerkinElmer Spectrum 3™) in the range 4000–600 cm^–1^.

### 2.6. Determination of changes in fatty acid composition

To extract *H. rhamnoides* seed oil after microencapsulation, the microcapsules were added to hexane, fully dissolved, and sonicated. The supernatant was obtained by centrifugation. The n-hexane in the supernatant was evaporated to obtain the microencapsulated *H. rhamnoides* seed oil.

Based on the subject group’s previous research, the fatty acid methyl esterification was determined using the un-encapsulated *H. rhamnoides* seed oil or microencapsulated oil (0.50 mg), which was completely dissolved in 0.50 mL of NaOH-CH_3_OH solution (0.50 mol/L) at 60°C. Then, 0.50 mL of boron trifluoride-methanol solution was added and the reaction was carried out for 5 min at 80°C. The reacted sample was naturally cooled to room temperature, and 2.5 mL of hexane was then added under vigorous shaking. The mixture was left to stand, and the supernatant was collected for gas chromatography-mass spectrometry (GC-MS) analysis.

For gas chromatography, qualitative analysis of the fatty acid methyl esters in *H. rhamnoides* seed oil was performed on an Agilent 7890B-5977A gas chromatograph (GC-MS) coupled with a DB-FastFAME (60 m × 0.250 mm, 0.25 μm) column. The carrier gas flow rate was 1 mL/min. The initial column temperature was 80°C, and the temperature program is presented in Table 2. The injection volume was 1.0 μL, and the split ratio was 10:1. The conditions for mass spectrometry were set as a solvent delay of 5 min and a scan range of 40–500 m/z.

**TABLE 2:** Gas phase temperature rise procedure for GC-MS.

Heating rate (°C/min)	Temperature (°C)	Holding time (min)
	80	5
80	160	3
5	185	5
7	210	10
1	220	3
5	230	5

The fatty acid compositions in *H. rhamnoides* seed oil and microencapsulated oil were compared by matching the mass spectral searches with the NIST 20 mass spectrometry library, and the relative percentages of fatty acids were calculated by the peak area normalization.

### 2.7. Scanning electron microscopy (SEM)

The surface morphology of the microcapsules was observed through SEM, at an accelerating voltage of 15.0 kV, by photographing a uniformly distributed field of view.

### 2.8. Thermal stability analysis

The microcapsules (3–5 mg) were placed in a small crucible, and the temperature range of the differential thermal analyzer was set to 33–600°C, with a temperature rise rate of 10°C/min and nitrogen as the carrier gas, input at a rate of 20 mL/min. Thermogravimetric (TG) and differential scanning calorimetry (DSC) analysis of microcapsules were performed using NETZSCH STA 449 F3 Jupiter^®^.

### 2.9. Oxidation induction

The oxidation induction time of the un-encapsulated *H. rhamnoides* seed oil and the *H. rhamnoides* seed oil microcapsules was examined following the experimental method of a previous study (19). Weigh 0.60 g of microcapsules or oil into the test tube of the grease oxidation stability analyser (Wantong 892 professional oil oxidation stability tester), set the accelerated oxidation temperature at 120°C, the air flow rate at 20 L/h, the receiving pool of pure water 60.0 g, measure and record the oxidation induction time.

### 2.10. Comparison of antioxidant activity before and after microencapsulation

According to Zahra Akbarbaglu (24) et al. the active ingredients of the core material may lose their biological activity after exposure to high temperatures during spray drying. Therefore, the antioxidant activity of the raw *H. rhamnoides* seed oil (5.0, 4.0, 2.0, 1.0, 0.2 mg/mL) and microcapsules containing the same amount of seed oil was compared. Free radical scavenging assays were employed, including DPPH and ABTS assays.

#### 2.10.1. DPPH free radical scavenging assay

A DPPH solution was prepared at a concentration of 2.0 × 10^–4^ mol/L using anhydrous ethanol as the solvent. In tube 1, the DPPH solution was mixed with the seed oil solution to be tested (1:1, *v/v*); in tube 2, the solvent and DPPH solution (1:1, *v/v*) were mixed; in tube 3, the solvent and seed oil solution were mixed at 1:1 (*v/v*). The absorbance values of the three tubes were measured at 517 nm after 30 min of reaction and recorded as A, B, and C, respectively. The clearance rate was calculated as follows:


$$c\,l\,e\,a\,r\,a\,n\,c\,e\,r\,a\,t\,e\%=\frac{\left(B+C\right)-A}{B}\times 100\%$$

#### 2.10.2. ABTS free radical scavenging assay

ABTS (38.41 mg) was dissolved in 10 mL of water, and 66.20 mg of K_2_S_2_O_8_ was dissolved in 100 mL of water. Equal volumes of the ABTS and K_2_S_2_O_8_ solutions were mixed, and the reaction was carried out at 4°C in the dark for 12–16 h to obtain the mother liquor. The mother liquor was diluted 25 times with ethanol to obtain the ABTS working solution. The seed oil to be evaluated was mixed with the ABTS working solution (1:4 *v: v*) in tube 1, and water was combined with the ABTS working solution in tube 2. The reactions in tube 1 and 2 were carried out at room temperature for 30 min in the dark. The absorbances for the two tubes at 734 nm were measured as A and A_0_, respectively. The clearance rate was calculated as follows:


$$c\,l\,e\,a\,r\,a\,n\,c\,e\,r\,a\,t\,e\%=\frac{A_{0}-A}{A_{0}}\times 100\%$$

### 2.11. Data analysis

All data were obtained from three parallel experiments, and the results are expressed as the mean ± standard deviation. Data were processed using Excel and Origin 9.0, and the significance of the differences among multiple groups was analyzed using Duncan’s test.

## 3. Results and discussion

### 3.1. Selection of wall materials

The EEs and yields of microcapsules from the five wall combinations were obtained, and the results are presented in Table 3. The SPI:SPS combination had the highest EE and yield at 63.55 ± 4.52% and 57.00 ± 5.69%, respectively. The emulsions prepared with WPI:CS and WPI:MD showed delamination during resting and consequently, their EEs and yields were lower than those for the SPI:SPS combination. Furthermore, the emulsion prepared with SPI:GA had a high viscosity and the emulsion prepared with SPI:MD adhered to the inner wall during the spray-drying process, making both combinations non-conducive to spray drying. Therefore, the wall combination of SPI and SPS with a high EE and yield was selected as the optimal wall material combination. Similar to Fei-Ping Chen’s findings, SPI and SPS as wall materials can effectively encapsulate the core material (25).

**TABLE 3:** Encapsulation efficiencies (EEs) and yields of microcapsules with different wall combinations.

No.	Wall material and proportion (A:B)	Ratio (A:B)	EE %	Yield %
1	WPI:CS	1:2	47.32 ± 3.21	42.93 ± 4.34
2	WPI:MD	1:2	50.03 ± 2.43	42.67 ± 3.22
3	SPI:SPS	1:2	63.55 ± 4.52	57.00 ± 5.69
4	SPI:GA	1:2	51.03 ± 3.65	49.18 ± 2.47
5	SPI:MD	1:21	57.58 ± 2.87	31.12 ± 1.95

### 3.2. Process optimization

A single-factor method was used to prepare the microcapsules. The wall ratio (SPI:SPS), wall concentration, core-to-wall ratio, and spray-drying inlet temperature were investigated in terms of EE and yield to identify the best preparation process.

#### 3.2.1. Wall ratio (SPI:SPS)

**FIGURE 2:**
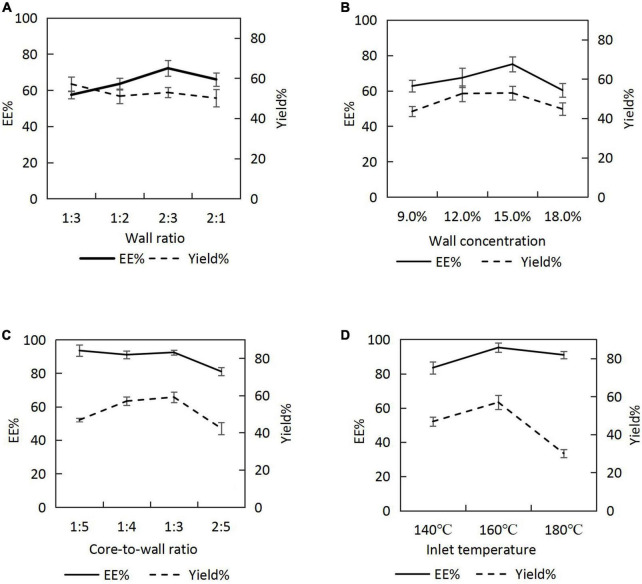
Encapsulation efficiencies (EEs) and yields of microcapsules under different process conditions: **(A)** wall ratio; **(B)** wall concentration; **(C)** core-to-wall ratio; **(D)** inlet temperature.

When the wall concentration was fixed at 9.0%, and the core-to-wall ratio was fixed as 2:5, the emulsion was prepared by varying the wall ratio (SPI:SPS = 1:3, 1:2, 2:3, and 2:1), and *H. rhamnoides* seed oil microcapsules were prepared at a spray-drying inlet temperature of 160°C. The EEs and yields are shown in Figure 2A. As the percentage of SPI increased, EE first increased and then decreased. The positive emulsification of SPI could effectively stabilize the oil, resulting in a high EE. The maximum EE of the microcapsules was achieved when the SPI:SPS was 2:3. However, as SPI continued to increase, the emulsion became more viscous, resulting in poor spray-drying results.

#### 3.2.2. Wall concentration

Emulsions were prepared by varying the wall concentration (9.0, 12.0, 15.0, and 18.0%) at a core-to-wall ratio of 2:5 and a wall ratio of 2:3, and the seed oil microcapsules were prepared at a spray-drying inlet temperature of 160°C. The EEs and yields (Figure 2B) increased and then decreased with an increasing wall concentration, reaching a maximum at the wall concentration of 15.0%.

#### 3.2.3. Core-to-wall ratio

The emulsion was prepared at a wall ratio of 2:3 and a wall concentration of 15.0%. The core-to-wall ratios were varied (1:5, 1:4, 1:3, and 2:5), and the spray-drying inlet temperature was 160°C. The EEs were > 90.0% for core-to-wall ratios between 1:5 and 1:3 (i.e. the oil contents of the microcapsules were between 16.67 and 25.00%) and decreased for a core-to-wall ratio of 2:5. When the seed oil content in the emulsion was increased, the wall could not wrap the core completely, resulting in a lower EE. Therefore, considering the oil content and the EEs of the microcapsules, 1:3 was chosen as the optimal core-to-wall ratio (Figure 2C).

#### 3.2.4. Inlet temperature

The *H. rhamnoides* seed oil emulsion was prepared at a wall ratio of 2:3, a wall concentration of 15.0%, and a core-to-wall ratio of 1:3. The microcapsules were prepared by varying the inlet temperatures to 140, 160, and 180°C during spray drying. The EEs and yields gradually increased with increasing inlet temperature (Figure 2D). However, when the inlet temperature reached 180°C, the EEs and yields decreased, possibly because of the rapid evaporation of water from the emulsion droplets at that temperature, which caused crusting, wrinkling, and cracking on the surface of the microcapsules. Subsequently, the optimal temperature for *H. rhamnoides* seed oil microencapsulation was identified to be 160 °C.

#### 3.2.5. Optimum conditions

Based on the EE and yield, the optimum preparation parameters were determined as a wall ratio for SPI:SPS of 2:3, a wall concentration of 15.0%, a core-to-wall ratio of 1:3, and an inlet temperature of 160°C. Under these conditions, the EE of *H. rhamnoides* seed oil microencapsulation was 95.30 ± 2.67%, and the yield was 57.03 ± 3.71%.

### 3.3. Effect of preparation process on the particle size of microcapsules

An important indicator for assessing the quality of microcapsules is the particle size. The smaller the particle size, the easier the release of the encapsulated material (26). Variations in median particle size (D50) of microcapsules under different process conditions are discussed below.

#### 3.3.1. Wall ratio

**FIGURE 3:**
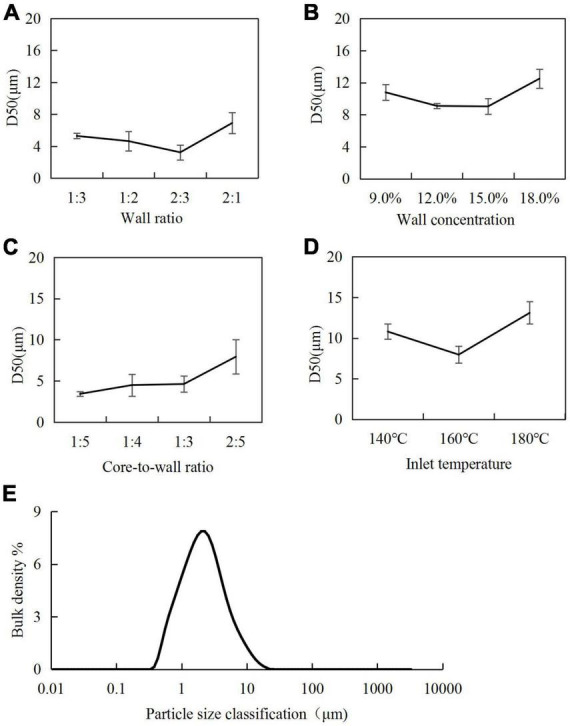
Particle size of microcapsules under different process conditions: **(A)** wall ratio; **(B)** wall concentration; **(C)** core-to-wall ratio; **(D)** inlet temperature; **(E)** particle size distribution of microcapsules under optimal preparation process.

The effect of the wall ratio in the range of 1:3–2:1 on the D50 was examined, and the results are shown in Figure 3A. When the SPI content was low, the poor emulsification and dispersion of *H. rhamnoides* seed oil resulted in a large D50. When the SPI content was too high, the emulsion viscosity increased, and the microencapsulated powder agglomerated during the spray-drying process, leading to an increase in D50. The smallest particle size was achieved when the SPI:SPS ratio was 2:3.

#### 3.3.2. Wall concentration

A wall concentration in the range of 9.0–18.0% was used to investigate its effect on the D50 of microcapsules (Figure 3B). The smallest particle size was achieved at a wall concentration of 15.0%. A higher concentration of the wall material increased the viscosity of the emulsion, leading to the agglomeration of the microencapsulated powder during the spray-drying process.

#### 3.3.3. Core-to-wall ratio

For core-to-wall ratios, the D50 of the *H. rhamnoides* seed oil microcapsules increased gradually with increasing core-to-wall ratio. Considering the oil content in the microcapsules, the optimal core-to-wall ratio was 1:3 (Figure 3C).

#### 3.3.4. Inlet temperature

For the inlet temperature, when the inlet temperature was 140°C, the slow loss of water during the drying process resulted in larger microcapsules. When the inlet temperature increased to 180°C, the surface of the microcapsules cracked, and some of the seed oil leaked and coalesced, leading to increased particle sizes. When the inlet temperature was 160°C, the microcapsule particles generated had the optimal size (Figure 3D).

#### 3.3.5. Optimum conditions

Considering the EEs, yields, and D50 of the microcapsules, the optimal conditions were determined as an SPI:SPS wall combination at a ratio of 2:3; wall concentration in the emulsion of 15.0%; core-to-wall ratio of 1:3 (25.0% oil content); and inlet temperature for spray drying of 160°C. The D50 of microcapsules prepared under these conditions was 7.96 ± 1.04 μm, and uniformity of dispersion (Figure 3E).

### 3.4. FT-IR analyses

**FIGURE 4:**
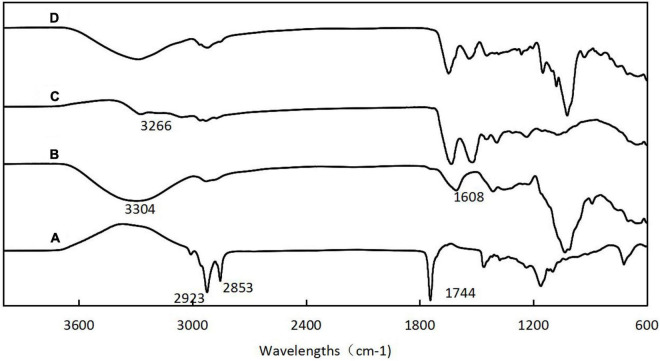
Fourier transform infrared spectroscopy (FT-IR) spectra of *H. rhamnoides* seed oil (A); SPS (B); SIP (C); *H. rhamnoides* seed oil microcapsules (D).

Figure 4 shows the infrared spectra of *H. rhamnoides* seed oil, SPS, SIP, and microcapsules. The peaks for seed oil at 2923 and 2853 cm^–1^ were caused by the C-H stretching vibration in the fatty acid carbon chain, and the absorption peak at 1744 cm^–1^ was caused by the stretching vibration of C = O in the fatty acid. The characteristic peaks of SPS were noted at 3304 cm^–1^, caused by the stretching vibration of O-H, and at 1608 cm^–1^, caused by the absorption peaks of the C = O in aldehydes and ketones. The characteristic peak for SIP was at 3266 cm^–1^, caused by a combination of -OH and N-H. A comparison of the spectrograms of the microcapsules showed that the characteristic peaks of the seed oil became weaker or even disappeared, indicating that the oil was well encapsulated within the wall material, thus reducing the movement of the *H. rhamnoides* seed oil molecules. The characteristic peaks of SPS and SPI showed different degrees of shift, indicating the formation of hydrogen bonds, Van der Waals hydrophobicity, and electrostatic forces among SPS, SIP, and seed oil, further corroborating that *H. rhamnoides* seed oil was encapsulated within the wall and formed efficient microcapsules.

### 3.5. Comparison of the fatty acid composition

**FIGURE 5:**
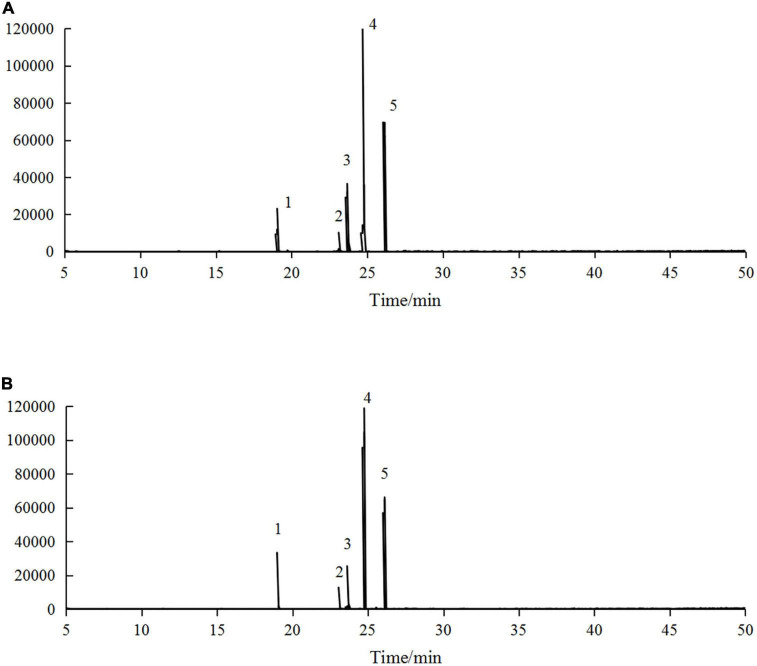
Gas chromatography-mass spectrometry (GC-MS) profiles of panel **(A)** *H. rhamnoides* seed oil and **(B)** microencapsulated *H. rhamnoides* seed oil: 1. palmitic acid methyl ester, 2. stearic acid methyl ester, 3. oleic acid methyl ester, 4. linoleic acid methyl ester, 5. α-linolenic acid methyl ester.

*Hippophae rhamnoides* seed oil contains a large amount of UFAs, and the loss or incompleteness of fatty acids is a key factor limiting its microencapsulation (27). Therefore, it is necessary to investigate the changes in fatty acids before and after microencapsulation. The GC-MS profiles of *H. rhamnoides* seed oil and microencapsulated oil are shown in Figure 5.

The main fatty acid compositions of *H. rhamnoides* seed oil and microencapsulated oil are presented in Table 4. There were five main fatty acid components in *H. rhamnoides* seed oil, i.e. palmitic acid (10.69 ± 0.06%), stearic acid (3.33 ± 0.16%), oleic acid (22.06 ± 0.25%), linoleic acid (53.42 ± 0.02%), and α-linolenic acid (9.05 ± 0.01%). Saturated fatty acids and UFAs accounted for 14.02 ± 0.22% and 84.53 ± 0.44%, respectively. In comparison, the UFAs in the microencapsulated oil were 84.57 ± 0.56%. This indicated that the fatty acid composition did not change significantly after microencapsulation, and that the SPI:SPS combination could effectively encapsulate *H. rhamnoides* seed oil.

**TABLE 4:** Fatty acid composition of original and microencapsulated *Hippophae rhamnoides* seed oil.

No.	Fatty acid	Relation time/min	Relative content in original oil (%)	Relative content in microencapsulated (%)
1	Palmitic acid (C16:0)	19.017	10.69 ± 0.06	10.72 ± 0.07
2	Stearic acid (C18:0)	23.105	3.33 ± 0.16	3.30 ± 0.04
3	Oleic acid (C18:1)	23.620	22.06 ± 0.25	22.02 ± 0.20
4	Linoleic acid (C18:2)	24.650	53.42 ± 0.02	53.42 ± 0.21
5	α-linolenic acid (C18:3)	26.047	9.05 ± 0.01	9.13 ± 0.15

### 3.6. SEM

**FIGURE 6:**
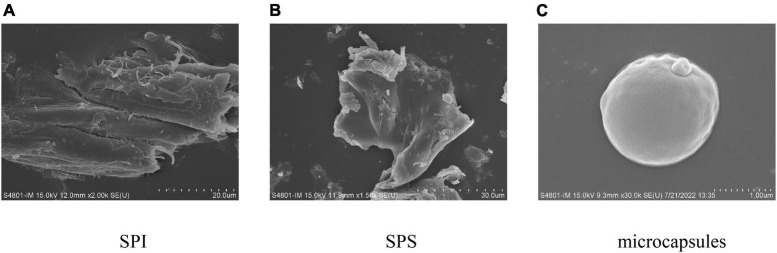
Scanning electron micrograph of panel **(A)** SPI, **(B)** SPS, **(C)** microcapsules.

Soy protein isolate, SPS, and the external morphology of the microcapsules prepared under optimal embedding conditions are shown in Figure 6. As can be seen from Figures 6A, B, both SPI and SPS have an irregular lamellar morphology. The microcapsules prepared by spray drying were spherical and structurally intact. The surface showed slight irregular depressions, probably because of the rapid evaporation of water from the particles during the spray drying process and the damage to the internal crystal structure of the microcapsules. The surface of the spray-dried droplets was dry and free from oil seepage, indicating that the *H. rhamnoides* seed oil was completely encapsulated within the wall material without cracked or hollow areas, indicating that the wall material could act as an effective protective layer.

### 3.7. Thermal stability analysis

**FIGURE 7:**
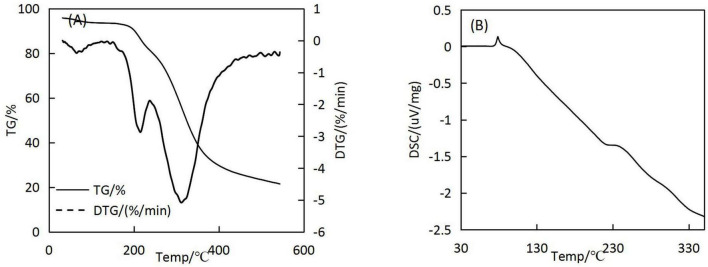
TG-DTG **(A)** and DSC **(B)** curves of microcapsules.

The results of the TG analysis of the microcapsules are shown in Figure 7A. The first stage of the decomposition of microcapsules occurred prior to 187°C, where the mass decreased by 7.91%. The second stage of weight loss occurred at 187–237°C, during which the microcapsules’ surface structure was disrupted and oil was gradually brought into contact with the external environment. The third stage of microcapsule breakdown occurred at 237–421°C; most of the microcapsules had degraded by this stage.

The results of the differential scanning calorimetry (DSC) analysis are shown in Figure 7B. Prior to 72°C, the DSC curve basically did not change, indicating that the structure of the microcapsules was stable did not change at this time. A heat absorption peak was observed at 78°C, possibly because of the denaturation of the wall material due to thermal swelling at high temperatures, resulting in a shift from an ordered crystal structure to a disordered crystal structure. When the temperature reached 105°C, the microcapsules dissolved completely, indicating that the *H. rhamnoides* seed oil microcapsules can maintain good thermal stability up to 78°C during regular use without the core material being affected.

### 3.8. Induced oxidation time of microcapsules

The oxidation induction times of *H. rhamnoides* seed oil and microcapsules were determined using an oil oxidation stability tester. The seed oil had an oxidation induction time of 2.13 ± 0.31 h. After microencapsulation, the time for oxidation induction was extended to 7.40 ± 0.47 h, under accelerated oxidation conditions, the oxidation induction time of microencapsulated *H. rhamnoides* seed oil was 2 times longer than that of unencapsulated, suggesting that the SPI:SPS combination could act as a barrier to protect *H. rhamnoides* seed oil from oxidation and increase shelf life. It is an effective means of improving the oxidative stability of *H. rhamnoides* seed oil.

### 3.9. Comparison of antioxidant activity

**FIGURE 8:**
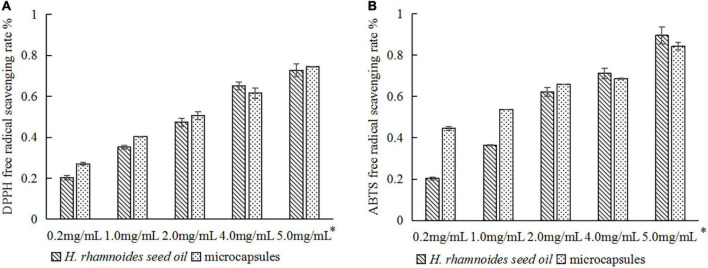
Comparison of antioxidant activity of *H. rhamnoides* seed oil and microcapsules: **(A)** DPPH free radical scavenging rate; **(B)** ABTS free radical scavenging rate. *Actual concentration of *H. rhamnoides* seed oil.

Figure 8 compared the DPPH and ABTS radical scavenging capacities of the raw *H. rhamnoides* seed oil and microencapsulated oil. There were no significant differences in the antioxidant activity at the same oil concentration, which is consistent with the findings of Xing Xin (22) and Tavani Rocha Camargo et al. (28). This indicates that micro-encapsulation maintains the antioxidant activity of *H. rhamnoides* seed oil and stabilizes the biological activity of the oil during processing and storage. Therefore, using a combination of SPI:SPS as a wall material can be considered an effective method to protect the bioactivity of *H. rhamnoides* seed oil.

## 4. Conclusion

This study evaluated the processing conditions to prepare *H. rhamnoides* seed oil microcapsules using the spray-drying method. Under the optimum conditions, i.e., a wall combination of SPI:SPS = 2:3, a wall concentration of 15.0% in the emulsion, a core-to-wall ratio of 1:3 (25.0% oil content), and an inlet temperature of 160 °C during spray drying, the EE and yield were 95.30 ± 2.67% and 57.03 ± 3.71%, respectively, with a D50 of 7.96 ± 1.04 μm. FTIR showed that seed oil was successfully encapsulated by the wall material. In addition, the composition of *H. rhamnoides* seed oil before and after encapsulation was analyzed by GC-MS and it was found that microencapsulation had little effect on the fatty acid composition of seed oil. SEM analyses showed that the surface of the microcapsules was smooth, with an irregular shape. TG and DSC analyses showed that the thermal stability of the microcapsules was good. Induced oxidation time and the scavenging ability showed that using the SPI:SPS combination as a wall material for encapsulation is an effective method to protect the biological activity and extend the shelf life of *H. rhamnoides* seed oil.

## Data availability statement

The original contributions presented in this study are included in the article/supplementary material, further inquiries can be directed to the corresponding author.

## Author contributions

LL: conceptualization and writing – review and editing. MG and WZ: methodology. HZ and GS: formal analysis. WM and XL: investigation. GS and DY: data curation. HZ: writing – original draft preparation. MG: supervision. All authors have read and agreed to the published version of the manuscript.
